# One Health Surveillance System in Gujarat, India: A Health Policy and Systems Research Protocol for Exploring the Cross-Sectoral Collaborations to Detect Emerging Threats at the Human-Animal–Environment Interface

**DOI:** 10.3390/tropicalmed8090428

**Published:** 2023-08-29

**Authors:** Sandul Yasobant, Deepak Saxena, Ravina Tadvi, Zahiruddin Quazi Syed

**Affiliations:** 1 Center for One Health Education, Research & Development (COHERD), Indian Institute of Public Health, Gandhinagar 382042, India; ddeepak72@iiphg.org (D.S.); rtadvi@iiphg.org (R.T.); 2 School of Epidemiology & Public Health, Datta Meghe Institute of Medical Sciences (DMIMS), Wardha 442004, India; 3 Global Health, Institute for Hygiene and Public Health (IHPH), University Hospital Bonn, Bonn 53127, Germany; 4 Global Consortium for Public Health Research, Jawaharlal Nehru Medical College, Datta Meghe Institute of Higher Education and Research (DU), Wardha 442004, India; zahirquazi@dmimsu.edu.in

**Keywords:** One Health, One Health surveillance systems, cross-sectoral collaborations, Gujarat, India

## Abstract

The close interaction between humans, animals and the ecosystem has been a reason for the emergence and re-emergence of zoonotic diseases worldwide. Zoonoses are estimated to be responsible for 2.5 billion human illnesses and 2.7 million deaths worldwide. Gujarat is a western state in India with more than 65 million people and 26 million livestock, and includes surveillance systems for humans and animals; however, more evidence is needed on joint collaborative activities and their effect on the early warning response for zoonoses. Thus, this study aims to investigate sectoral collaborations for early warning and response systems for emerging and re-emerging zoonoses, aiming to develop a One Health surveillance (OHS) system in Gujarat, India. This case study uses policy content analysis followed by qualitative and quantitative data collection among state- and district-level surveillance actors to provide insight into the current cross-sectoral collaborations among surveillance actors. It helps identify triggers and documents factors helpful in strengthening cross-sectoral collaborations among these systems and facilitates the establishment of an OHS system in Gujarat, India.

## 1. Introduction

### 1.1. Global and Indian Burden of Emerging and Re-Emerging Zoonotic Diseases

Evidence indicates an increasing burden from recent pandemics of emerging and re-emerging zoonotic diseases attributed to complex linkages threatening to the human–animal–ecosystem [1,2]. Emergence and re-emergence of zoonotic diseases refers to the occurrences of new or previously known infections transmitted from animals to humans, which have significant public health implications and may lead to outbreaks or epidemics [3]. It is estimated that zoonoses are responsible for 2.5 billion cases of human illness and 2.7 million human deaths worldwide each year [4]. India is not an exception to the global burden with significant public health zoonotic diseases such as rabies, brucellosis, toxoplasmosis, cysticercosis, echinococcosis, Japanese encephalitis (JE), plague, leptospirosis, scrub typhus, Nipah virus disease, trypanosomiasis, Kyasanur forest disease (KFD), and Crimean-Congo haemorrhagic fever. According to an International Livestock Research Institute study, 13 zoonoses cause 2.4 billion cases of human illness and 2.2 million deaths yearly [5]. 

### 1.2. Importance of Early Warning and Surveillance Systems: The Role of the One Health Approach 

Rapid response and implementation have been vital in limiting the spread of zoonotic diseases, such as the Nipah virus outbreak in Kerala in 2018 [6]. The rapid outbreak of emerging or re-emerging zoonotic diseases requires an effective surveillance system to take timely control measures [7]. Current routine surveillance is focused on known diseases and clinical syndromes. Still, the increasing likelihood of emerging disease outbreaks shows the critical importance of early detection of unusual illnesses or the circulation of pathogens—before human disease manifestation [8]. Early detection is essential to trigger a timely disease outbreak investigation and to reduce the outbreak’s impact by minimising mortality or morbidity [9]. Among others, the Global Early Warning System (GLEWS) is an example of a global information system, which is a joint effort between the Food and Agriculture Organization (FAO), the World Organization for Animal Health (WOAH), and the World Health Organization (WHO), bringing together human/veterinary public health systems to share zoonotic disease outbreak information and epidemiological and risk analysis [10]. Thus, a future catch-all infrastructure would not be limited to surveillance based solely on human clinical cases, but would also need to improve the generation and access to data from other reservoirs, as per the One Health principles [11]. One Health emphasises the multi- and/or transdisciplinary actions that require collaboration among various actors in dealing with disease control or risk mitigation and promoting the health and well-being of humans, animals, and the environment to improve efficiency and effectiveness in managing health threats [11]. Not only for the sustainable management of zoonotic diseases [12] but also for the prevention and control of these zoonotic diseases [13], the One Health approach is found to be the most appropriate [4,13,14]. One Health surveillance (OHS) describes the systematic collection, validation, analysis, interpretation and dissemination of information collected on humans, animals and the environment to inform decisions for more effective, evidence- and system-based health interventions. The critical element of One Health surveillance is collaboration in planning, coordinating and implementing central functions across a wide range of sectors and disciplines. However, there is a lack of evidence on how to convert the current surveillance systems to the OHS. 

### 1.3. Global Action for Cross-Sectoral Collaboration to Strengthen Surveillance and EWRS

The WHO defines the early warning response system (EWRS) as a system that provides an early warning of acute public health events and then connects this function to an immediate public health response, an essential part of the surveillance system [15]. There were noteworthy initiatives like the Global Early Warning and Response System for Animal Diseases including Zoonoses (GLEWS) at the international level between WOAH, WHO and FAO indicating cross-sectoral collaborations [16]. Another noteworthy initiative is Human Animal Infections and Risk Surveillance (HAIRS), an exemplary example of the cross-government horizon, which has worked across several organisations since 2004. Even national-level initiatives, such as in Thailand, have successfully implemented One Health surveillance since 2001 to prevent and control the Nipah virus among bats, humans and pigs through collaboration with various departments [17]. Guinea, Liberia and Sierra Leone have also set an exceptional example of collaboration by developing a national One Health platform to prevent public health threats [18]. However, all these initiatives focus on cross-sectoral data-sharing across the surveillance system; there needs to be more understanding of implementing the same in different health system structures.

### 1.4. Cross-Sectoral Collaborations across the Surveillance Systems in the Context of Gujarat, India

Gujarat is in western India, with more than 63 million people (as of 2021) and 26 million livestock (as of 2020) [19,20]. Gujarat is facing more significant focal outbreaks of Crimean-Congo haemorrhagic fever, avian influenza, chikungunya, Zika, etc. [21,22]. To tackle this emergence of zoonoses, Gujarat has an ongoing, systematic and timely collection of data from various sources (disease occurrence, distribution, determinants of transmission) for analysis, interpretation and dissemination to relevant stakeholders for action in both human and animal health systems. The human health system has an Integrated Disease Surveillance System (IDSP) under the Integrated Health Information Platform (IHIP), known as IHIP-IDSP [23], and the animal health system has a National Animal Disease Reporting System (NADRS) and a National Animal Disease Referral Expert System (NADRES) [24]. The surveillance systems have evolved to conduct surveillance within the domains of human health, animal health, and the environment but in silos and are tightly linked as per the respective governing feedback. To monitor the emergence of new zoonoses, a veterinary consultant was appointed under the IHIP-IDSP for the establishment of inter-sectoral coordination with the Departments of Animal Husbandry, Environment and Forest, and Agriculture to look at the data from NADRS and NADRES and to match those with IHIP-IDSP data to compile them all on one platform, thereby supporting the effective operational integration of disease control efforts based on the surveillance data [25]. Despite the need to establish inter-sectoral coordination between these surveillance systems for generating early warning signals (EWSs), joint investigations and responses to outbreaks of zoonotic diseases are still the least functional in Gujarat. There are national and international warrants to establish a proactive, coordinated, interdisciplinary and cross-sectoral approach across human, animal, and environmental sectors, which remain the core pillar of the One Health framework, to mitigate the public health challenges [26,27]. The cross-sectoral collaborations have evidenced how these partnerships in disease surveillance responded to emerging public health threats in Kerala state, India [28]. However, in Gujarat, there needs to be more understanding of the cross-sectoral collaborations among these surveillance systems, as evidenced in the previous research [21]. The advantage of examining such collaborations from several sectors is that it allows for the impact of different perspectives at each level to be made clear, and also uncovers nuances and interactions between actors that may impact effectiveness. Thus, this study aims to investigate sectoral collaborations for the One Health surveillance system (OHS) in Gujarat, India and intends to answer questions about the level of collaboration among human/animal disease surveillance system actors for zoonotic disease prevention and control, and how to manifest cross-sectoral collaborations for early warning and the response to zoonotic diseases in Gujarat, India. 

## 2. Experimental Design 

### 2.1. Study Definitions

Table 1 indicates the operational definitions used in this study. 

### 2.2. Study Design

This explanatory case study comprises policy content analysis followed by primary qualitative and quantitative data collection in Gujarat, India, from 2023 to 2024. 

### 2.3. Conceptual Framework

**Figure 1:**
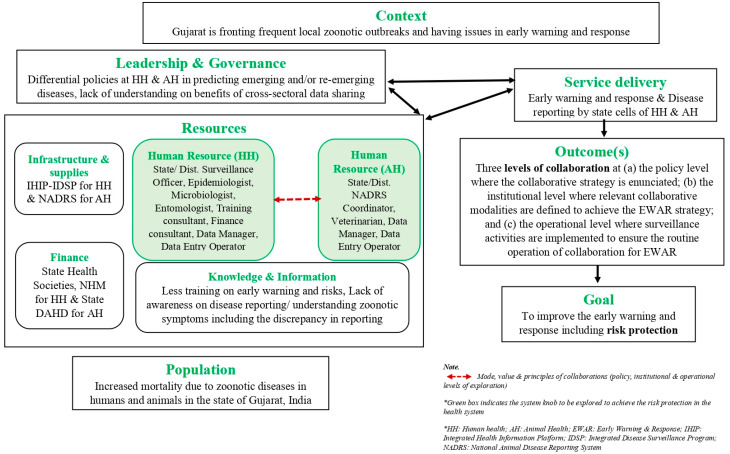
A conceptual framework for cross-sectoral collaborations across human and animal health surveillance actors adopted and modified from van Olmen J. et al., 2012 [37].

This research is conceptualized based on system thinking in public health. As many theories have been embedded within system thinking, for this research, we are adapting the general systems theory (GST), outlined by Ludwig Bertalanffy in 1969 [36]. Systems theory aims to systematically discover a system’s dynamics, constraints and conditions, and to elucidate principles that can be applied to systems at every level and in every field, to achieve optimised outcomes. Some systems function mainly to support other systems by aiding in maintaining the other systems to prevent failure. Changing one part of the system usually affects other parts or the whole system, with predictable behaviour patterns. For self-learning and self-adapting systems, growth and adaptation depend upon how well the system is adjusted to its environment. This study adopts the health system dynamics framework developed by van Olmen J. et al. [37] and modifies it to the context of One Health surveillance. Here, we assert that the surveillance unit and respective services like early warning and response and disease reporting are at the core, which rely on leadership, governance, position, and interactions with other actors. We acknowledge that the dynamics of interactions depend highly on the different blocks of the framework, as shown in Figure 1; this study will entertain only the human resource block and its soft-core values.

As indicated in Figure 1, the conceptual framework is adopted and modified by Olmen J. et al. to improve the EWR, including risk protection in Gujarat, India. To achieve this, there is a need for cross-sectoral collaborations at various levels, i.e., policy levels, institutional levels, and operational levels, for the timely collection of data, analysis, and communication. However, to understand the cross-sectoral collaborations from the HSPR perspective, resources, i.e., infrastructure and supplies, human resources, finance, and knowledge and information, are essential in addition to leadership and governance. This particular study focuses exclusively on the cross-sectoral collaborations across the human resources of the human and animal health surveillance system engaged at different levels (policy, institutional and operational). 

### 2.4. Study Setting 

**Figure 2:**
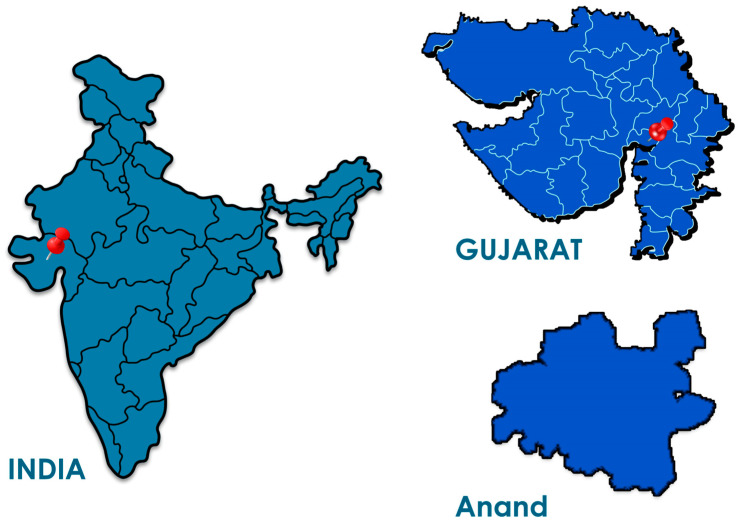
The study site indicating (**left**) Gujarat state in India, (**right top**) Anand district of Gujarat state and (**right bottom**) Anand district, Gujarat, India.

The proposed study will focus on two tiers: one at the state level (Gujarat, India) and the other at the district level (Anand district, Gujarat). Anand is known in the history of modern India because of the White Revolution; it also has some of the most significant cooperative-sector development, at a population of 2 million. Anand’s district level is considered in this study for two specific reasons: first, long-standing outbreaks have been documented in this area; and second, Anand is among cities with the highest risk of infectious disease outbreaks [38]. Also, the presence of the area’s large cooperative sector and dairy production supports investigating its human and animal health surveillance systems. Henceforth, both surveillance systems will be considered the OHS. Figure 2 indicates the study sites, Gujarat state and Anand district.

## 3. Materials and Equipment 

### 3.1. Content Extraction Tool

The content extraction aims to fit the criteria for the matrix to evaluate multisectoral collaboration for the OHS developed by Bordier M et al. [32]. The evaluation criteria for the matrix are based on the following: collaborative strategy, modalities, coverage, resources, steering and coordinating mechanisms, scientific and technical support, training, information, monitoring and evaluation, engagement, surveillance design, sampling, laboratory activities, data and result sharing, data stock management, data analysis and interpretation, internal and external communication, dissemination.

### 3.2. Primary Data Collection

Primary data will be gathered from both human and animal disease surveillance stakeholders from the state and district levels. The data collection will use qualitative and quantitative methods. 

#### 3.2.1. Qualitative Data Collection

A semi-structured interview guide aims to map the triggers from the respective surveillance systems, whether cross-sectoral collaborations materialised or not. Two situations will be emphasised: the first, during the last outbreak (from the individual health system), where joint actions were taken; the second during routine surveillance activities. The different levels of collaboration, i.e., environmental (social, economic and political forces, outside relevant stakeholders), organisational (orientation of commitment to goals, how members are organised, i.e., the intensity of linkages, power/authority, interdependence, the autonomy of respective sectors, coordination mechanisms), and operational (interaction among members, trust and reciprocity, perception, values, attitudes) will be captured for the two specified situations mentioned above. 

For documenting how to manifest the collaborations, the interview guide will also focus on prerequisites for collaboration emanating from policy outcomes and professional processes, particularly the level of integration between the sectors involved and the perceptions of collaboration. Further, it will also explore the trigger points that will lead to establishing a cross-sectoral collaboration and developing an OHS in Gujarat. This rationale for data capture will follow the principles of Williumsen E et al. [39].

#### 3.2.2. Quantitative Tool

A structured and validated questionnaire in the Indian context by Glandon D et al. [40] measuring the collaboration among healthcare workers will be administered to the surveillance actors. This Frontline Health Workers Multisectoral Collaboration (FLW-MSC) consists of 18 items on open communication, respect, help and support, role clarity, willingness to listen, joint planning, information sharing, trust, power sharing, shared vision, service coordination, enabling environment, accountability, conflict management, interdependence, commitment/motivation, training/guidance and leadership/incentives. Responses on these items will be collected on an ordinal scale (1–Never, 2–Seldom, 3–Sometimes, 4–Most of the time, and 5–Always). Furthermore, a specific questionnaire section on the type of interaction as part of understanding the health system network will also be collected to understand the surveillance network’s strength. The types of interaction will be used to measure the network’s strength in two chosen situations ranging from not linked (do not work together), communication (share information only), cooperation (working together informally to achieve common goals), collaboration (working together as a formal team with specific responsibilities, e.g., formal agreement), to fully linked (work together as a formal team; mutually plan and share staff or resources to accomplish goals). Here, we are interested in studying the complete networks, i.e., all surveillance actors with all dyads. We will adapt both free choices, i.e., stakeholders that are chosen from a given list; and free calls, i.e., stakeholders that are chosen unrestrictedly for documenting the interaction with different actors within the boundary [41]. 

## 4. Detailed Procedure

**Figure 3:**
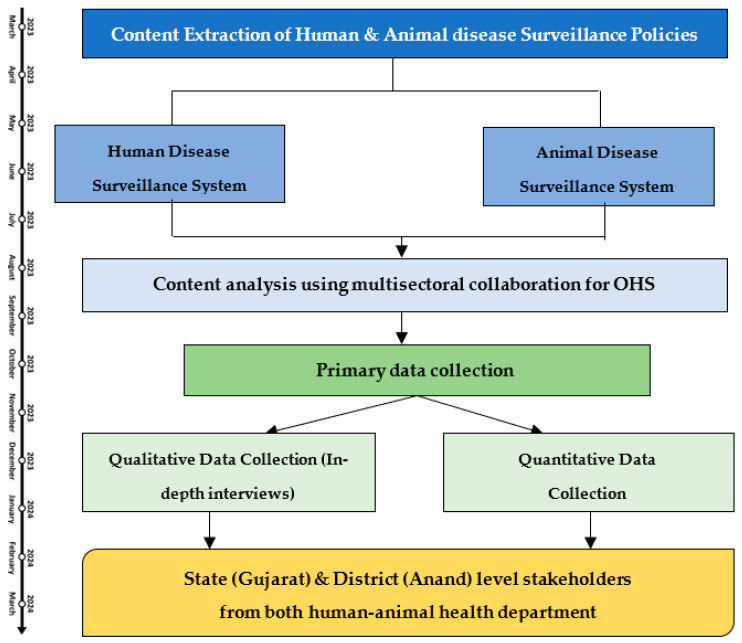
Flowchart of the detailed study procedure.

The detailed step-wise procedure of the study is illustrated in the following Figure 3.

### 4.1. Content Extraction and Analysis

Content extraction will identify and include human and animal health surveillance policies. Further, the selected zoonotic disease outbreak management guidelines will also be included for the extraction. A content extraction sheet will be prepared, and the relevant data, as per the criteria matrix, will be designed for analysis as recommended by Bordier M et al. [32]. A total of 75 criteria will be graded under 23 organisational attributes, 9 functional attributes, and 3 organisational indexes. For each criterion, four possible grades, ranging from 0 to 3, are possible, and a detailed definition of the situation according to which each grade should be awarded is provided in the scoring sheet. As per the recommendations, results will be expressed on a five-tiered scale, from A to E. Once the scoring is carried out, the spreadsheet automatically produces three graphical representations of the evaluation results in the third sheet. This visual layout shows the quality of the collaborative effort within the multisectoral surveillance system. Different chart types help to differentiate the three levels of evaluation easily obtained: organisation at a micro level, organisation at a macro level, and functions. This analysis will help to identify the specific collaborative functions that need to be strengthened to make the system more effective.

### 4.2. Primary Data Collection and Analysis 

#### 4.2.1. Qualitative 

In-depth interviews will be conducted with the actors purposely selected from both surveillance systems. The one-to-one interviews will be conducted at a convenient time for participants after obtaining their consent to participate in the study. An interview guide with broad, open-ended questions on the respondents’ collaboration with other actors during different health system situations will be captured. Audio recording and verbatim notes will be taken during the interviews.

Transcripts will be made from the interview recordings and field memos. Both inductive and deductive codes will be generated. Similar codes will be combined into themes of collaboration [42]. First, the three levels, i.e., environmental, organisational, and operational levels of collaboration; and second, the two situations, i.e., routine surveillance and during the last outbreak, will be emphasised in the analysis. At the environmental level, external legitimate network operations, key actors who are continuously supporting/influencing the state cells, a history of shared resources, etc., will be examined. Meanwhile, at the organisational level, understanding the goals of collaboration along with formal/informal rules/regulations, maintaining inter-professional relations, etc., will be examined. And at the operational level, experiences of working together, effective resolution of conflicts (if any), skills to carry out the collaborations, etc., will be focused on in the analysis. For the perception and ways to manifest the cross-sectoral collaborations, three differential thematic aspects, i.e., individual factors (work motivation, role expectations, personality, professional power), group factors (leadership, coping, communication, social support), and organisational factors (culture, domain, environment) will be analysed. To ensure that the results are a reflection of the data, the codes/themes will be related to the original data [43]. The findings will be reported using consolidated Criteria for Reporting Qualitative Research [44]. This analysis will help to understand the current situation and possibilities of convergence in the surveillance system. 

#### 4.2.2. Quantitative 

The quantitative data from the FLW-MSC assessment will be handled like any other Likert scale data. The continuous and categorical expressions will be used per the principles recommended by Glandon D et al. [40]. The descriptive statistics like mean, standard deviation for continuous variables, and proportion for qualitative variables will be generated through statistical software R version 3.4.1 [45]. We will adapt the network analysis for the network data to find the convergence points of human and animal health system surveillance actors with their strengths. Social network analysis (SNA) is defined as a distinctive set of methods used for mapping, measuring, and analysing the social relationships between people, groups, and organisations [46,47]. This analysis intends to provide insights into stakeholder relationships, especially to understand the dynamics within a health system [48] as SNA has proved to help understand the nature of relations between actors within a system and how these relationships influence the structure of a system [47,49]. A visualisation of the interactions and quantified outcomes such as average degree (the average number of links each node in the network has), density (the proportion of possible connections in the network), and degree of centralisation (the extent to which only a few nodes have a large number of ties) will be analysed through UCINET version 6 [50]. This analysis will help quantify the strength of the health system network. 

### 4.3. Study Limitations

This proposed study has several limitations. First, the data will be collected in a cross-sectional mode. Collecting live efforts on cross-sectoral collaborations during an ongoing outbreak might be ideal. However, this might also pose an impetus to be part of the system during any health emergency. Second, the number of stakeholders at the state level is fewer; thus, the network strength might not provide a comprehensive picture. Due to time constraints, this study only targets state-level stakeholders, not units above or below. Third, there might be a possibility of a negative outcome for this study where we end up with no cross-sectoral collaborations. However, this exercise will still provide insight into the preparedness of the human and animal health surveillance systems for developing a future One Health surveillance in the state of Gujarat.

## 5. Expected Result

This study involves the descriptive and explanatory arm; the findings will be triangulated during the analysis. The quantitative analysis will provide insight into the attributes of the current surveillance system. In contrast, the qualitative data will use these attributes to infer how to manifest the cross-sectoral collaborations for early warning response. All the quantitative and qualitative data will be triangulated at the end. This study will provide insight into the current levels of cross-sectoral collaboration for the OHS, which is essential for detecting emerging threats early and developing an early warning response system. Further, this will also assist in documenting the attributes for strengthening cross-sectoral collaborations among these systems. Additionally, the triggers for cross-sectoral collaboration will be one of the findings from this study. This exercise will be the first of its kind at the state level, boosting the programmatic performance and assisting in envisioning the OHS in Gujarat.

## Figures and Tables

**Table 1:** Operational Definitions.

Disease prevention [29]	Disease prevention is defined as specific, population-based and individual-based interventions for primary and secondary (early detection) prevention, aiming to minimise the burden of diseases and associated risk factors.
Disease control	Disease control is the reduction of disease incidence, prevalence, morbidity, or mortality to a locally acceptable level.
Disease surveillance system [30]	Disease surveillance is an information-based activity involving the collection, analysis and interpretation of large volumes of data from various sources.
Early warning system [31]	An early warning system is a warning system that can be implemented as a chain of information communication systems and comprises sensors, event detection and decision subsystems for the early identification of hazards.
One Health [11]	One Health is an integrated, unifying approach that aims to sustainably balance and optimise the health of people, animals and ecosystems. It recognises that the health statuses of humans, domestic and wild animals, plants, and the wider environment (including ecosystems) are closely linked and interdependent. The approach mobilises multiple sectors, disciplines and communities at varying levels of society to work together to foster well-being and tackle threats to health and ecosystems while addressing the collective need for clean water, energy and air, safe and nutritious food, action on climate change, and sustainable development.
One Health Surveillance [32]	One Health surveillance describes the systematic collection, validation, analysis, interpretation and dissemination of information collected on humans, animals and the environment to inform decisions for more effective, evidence- and system-based health interventions. The critical element of One Health surveillance is collaboration in planning, coordinating and implementing central functions across a wide range of sectors and disciplines.
Cross-sectoral collaborations [33,34]	In 1998, the Health Promotion Glossary was defined as “cooperation between different sectors of society, such as the public sector, civil society, and the private sector”. In 2008, it was defined as “actions undertaken by sectors outside the health sector, possibly, but not necessarily, in collaboration with the health sector, on health or health equity outcomes or the determinants of health or health equity.”
Levels of collaboration (environmental, organizational, operational) [35]	The environmental level refers to the impact of the external environment, including all relevant stakeholders surrounding the network and its operations. The organisational level refers to the effect of the structural characteristics of the different types of networks. The operating level relates to the interactions among the individual network participants.

## Data Availability

All the data from this study will be made available upon its completion. Authors who fulfil the open-access criteria feel free to reach the corresponding author for full access to data: Dr. Sandul Yasobant, Centre for One Health Education, Research & Development (COHERD), Indian Institute of Public Health Gandhinagar, Opp. Air-Force Head Quarters, Nr. Lekawada, 382042 Gandhinagar, Gujarat, India (email: yasobant@iiphg.org).
